# Evaluation of cancer testis antigen (CT10, PRAME) and MHC I expression in high grade urothelial carcinoma of the bladder

**DOI:** 10.1007/s00428-019-02661-2

**Published:** 2019-09-02

**Authors:** Anjelica Hodgson, Achim A. Jungbluth, Nora Katabi, Bin Xu, Michelle R. Downes

**Affiliations:** 1 Division of Anatomic Pathology, Department of Laboratory Medicine and Molecular Diagnostics, Sunnybrook Health Sciences Centre, 2075 Bayview Avenue, Toronto, ON, Canada; 2 Department of Laboratory Medicine and Pathobiology, University of Toronto, 1 King’s Colege Circle, Toronto, ON, Canada; 3 Department of Pathology, Memorial Sloan Kettering Cancer Center, 1275 York Avenue, New York, NY, USA

**Keywords:** Bladder cancer, urothelial carcinoma, inflammation, cancer testis antigens, major histocompatibility complex I, prognosis

## Abstract

Immunotherapeutic strategies are increasingly used in the treatment of a number of malignancies including high grade urothelial carcinoma (HGUC) of the bladder. Because of this, detailed and accurate assessment of the tumour immune microenvironment is paramount. In this study, we aimed to correlate the composition of the tumour immune microenvironment with oncologic outcome and the expression of two cancer testis antigens (CTAs), CT10 and PRAME, potential cancer vaccine targets, as well as major histocompatibility complex I (MHC I), a molecule associated with tumour immune escape and resistance to immunotherapy.

Triplicate tissue microarrays (TMAs) were constructed using 207 cases of HGUC of the bladder. Oncologic outcome data was gathered for each case. Consecutive sections from the TMA blocks were stained with CD3, CD4, CD8, FOXP3, PD1, PD-L1, CT10, PRAME, and MHC I. 21% and 15% of cases expressed CT10 and PRAME, respectively. 88% of cases showed absent or decreased MHC I expression. CT10-expressing tumours showed a significantly worse disease specific survival (p = 0.007, hazard ratio 2.245, confidence interval 1.223–4.122). CT10, PRAME, and MHC I expression significantly correlated with other some immune parameters. CT10 and PRAME are expressed in a subset of HGUC and CTA and MHC I expression correlate with a number of important immune parameters. Together, these findings highlight the potential for exploring novel immune therapeutic strategies in HGUC. Additional studies evaluating the clinical relevance of these findings are warranted.

## INTRODUCTION

**Fig 1:**
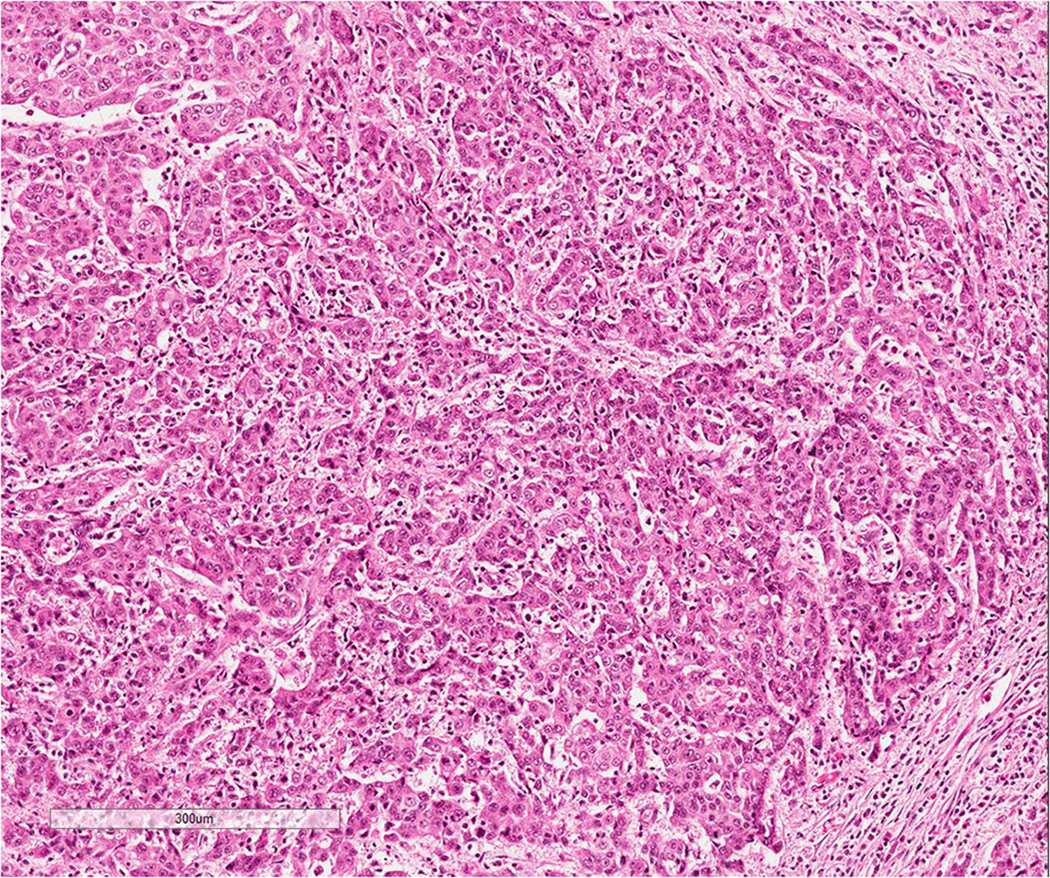
Example of high grade urothelial carcinoma; note the prominent inflammatory infiltrate

High grade urothelial carcinoma (HGUC) of the bladder (Figure 1) is a common and aggressive tumour type which is increasingly being studied with regards to its tumour immune microenvironment. Our group and others have previously shown that the composition and degree of inflammation surrounding and within a tumoural focus of HGUC may provide information about the tumour’s underlying biology[1] and likely prognosis[2–6].

In addition to therapy targeting programmed cell death 1 (PD-1)/programmed cell death ligand 1 (PD-L1) which is being used in the treatment of a number of malignancies including HGUC[7–12], cancer vaccines are another immunotherapy treatment strategy which is being studied in a number of tumour types. Cancer vaccines function to activate a patient’s immune system in order to mount an immune response specifically targeting cancer cells[13, 14] and importantly, some cancer vaccines have shown promising benefit in recent clinical trials[15]. One specific target for cancer vaccines is the cancer testis antigens (CTAs), a group of molecules found to be expressed in different cancer types and in normal tissues restricted to immune privileged site such as testicular germ cells[16]. Interestingly, in addition to potentially serving as targets for cancer vaccines, CTAs may play a role in modulating tumoural gene expression[17] and in supporting tumour cell growth and survival[18]. The expression of a number of CTAs in HGUC has been studied by some groups[19–21] including Sharma *et al.* who showed that 77% of the studied cases expressed at least one of nine CTAs while 61% of cases expressed two or more CTAs[22]. Preferentially Expressed Antigen in Melanoma (PRAME) is another CTA which has been shown to be highly upregulated in a number of different neoplasms[17]; interestingly, it has been suggested that the PRAME may antagonize retinoic acid receptor signalling, thereby conferring proliferative advantages to tumour cells[23].

Integral to the proper functioning of the normal immune system is Major Histocompatibility Complex I (MHC I), a cell surface protein complex present on all nucleated cells. MHC I normally functions to present “non-self” cytosolic peptide antigens to CD-8+ T cells, eventually leading to propagation of the cytotoxic pathway leading to cell death of malignant or virally infected cells. Because of its importance in the establishment of a cytotoxic response, lack of MHC I expression on tumour cells has not surprisingly been proposed as an important mechanism in tumour immune escape and resistance to immunotherapy[15, 24–26].

The aim of this work was to expand our understanding of the tumour immune contexture in HGUC of the bladder using an immunohistochemical approach. In light of the ongoing developments in the study of MHC I, CTAs and cancer vaccines, we chose to assess the association of CTAs with immune markers and impact on oncologic outcomes.

## MATERIALS AND METHODS

This project was approved by the Research Ethics Board at Sunnybrook Health Sciences Centre in Toronto, Ontario, Canada (REB 187–2016).

### Case selection and review

All cases were identified through a retrospective search of the laboratory information system, Sunquest CoPath (n=235), as previously described[27]. Briefly, the search criteria included cases of HGUC of the bladder treated by cystectomy between 1999 and 2015. Exclusion criteria included cases that were non-invasive, non-urothelial histology, presence of a neuroendocrine carcinoma component and divergent differentiation (squamous, glandular, sarcomatoid) encompassing > 50% of the tumour. Original hematoxylin and eosin (H&E) slides for each case were retrieved from our departmental archive and reviewed by a pathologist with subspecialty training in genitourinary pathology (MRD) who confirmed the tumour histology and grade.

The following demographic and clinicopathologic data was recorded for each case: age at diagnosis, patient sex, smoking status, tumour size, tumour focality, presence/absence of carcinoma in situ, presence/absence of lymphovascular invasion, margin status, presence/absence of lymph node metastases, history/type of neoadjuvant therapy (if applicable), pT stage, AJCC stage, date of last known follow up, date of disease relapse (if applicable), and date of death (if applicable). Evidence of disease relapse was based on operative and/or radiologic findings. Only deaths that occurred within the hospital or under the palliative care service were accessible.

### Tissue microarray construction

Triplicate 1 mm core tissue microarrays (TMAs) were constructed from 207 of the available cases; 28 cases were excluded from the TMAs due to lack of tissue or when a suitable tumour block for punching was not available. Technical details regarding construction of the TMAs have been previously described[27]. 4-micron thick unstained sections were prepared from the TMA blocks and were stained with CD4, CD8, FOXP3, PD-1, and PD-L1 (SP263) in a sequential fashion; CT10, PRAME, and MHC I staining was completed on a different date on unstained sections from the same TMA blocks.

### Immunohistochemistry and scoring

Immunohistochemical expression was assessed in immune cells (ICs) and/or tumour cells (TCs), depending on the marker being evaluated. The identification of TCs and ICs was based on morphologic features alone with assistance of H&E slides. All scoring was done by a single reviewer who is subspecialist genitourinary pathologist (MRD).

CD4, CD8, FOXP3, and PD-1 expression was assessed in ICs (CD4, CD8, PD-1 – membranous, FOXP3 – nuclear). For each marker, the absolute number of positive cells was evaluated in one hot spot/core (i.e. 1 representative 40x field/core), with the results averaged across all cores per case. PD-1-positive cases were dichotomized into high and low groups based on the median value of positive cells/40x field. PD-L1 expression (SP263 clone, Ventana Medical Systems, AZ, USA) was assessed in ICs (cytoplasmic or membranous staining of any intensity) and TCs (partial or complete membranous staining). The Ventana Benchmark Ultra automated staining platform was utilized according to the manufacturer’s protocol with the Optiview DAB IHC detection kit. TCs and ICs staining was assessed as present or absent with the estimated percentage of positive TCs and positive ICs recorded for each TMA core. The results were then averaged across the triplicate cores to give one estimate of percentage TCs and percentage ICs for each case. The degree of PD-L1 staining was considered positive when > 25% of TCs or ICs showed expression. MHC I expression (A4 clone, ThermoFisher Scientific, Waltham, MA, USA) was assessed in TCs; the percentage of TCs with cytoplasmic staining was noted and the cases were categorized as follows: positive (>75% of TCs), decreased expression (25–74% of TCs), or negative (< 25% of TCs). CT10 (CT10#5 clone, provided by the Ludwig Institute for Cancer Research, New York, NY, USA)[28] was assessed in TCs and any immunopositivity was considered positive. The location of staining (cytoplasmic and/or nuclear) and the percentage of CT10-positive TCs was recorded. Like CT10, PRAME (EPR20330 clone, Abcam, Cambridge, MA, USA) immunopositivity was defined as any expression within TCs. The location of staining (cytoplasmic and/or nuclear) and the percentage of PRAMEpositive TCs was also recorded.

### Statistical analysis

All statistical analyses were performed using the SPSS software 24.0 (IBM Corporation, New York, NY, USA). The prognostic significance of PRAME, CT10 and MHC I was assessed using log rank test with Kaplan Meier analysis for disease specific survival (DSS) and progression free survival (PFS). Hazard ratio and 95% confidence interval were calculated using Cox proportional regression model. The Pearson correlation coefficient was computed among the expression of each antibody. P values less than 0.05 were considered to be statistically significant.

## RESULTS

### Demographic and clinicopathologic characterization

The mean and median age at diagnosis was 70.3 and 71.0 years of age, respectively (range 33–93 years). Males (n = 150, 73%) were more commonly affected compared to females (n = 57, 27%). 59 (29%) of the patients had no smoking history, while 38 (18%) and 82 (40%) patients respectively were current or former smokers, respectively. Information regarding smoking status was not available for 28 (13%) patients.

131 (63%) tumours were more than 3 cm in size while 70 (34%) were less than 3 cm in size; 6 (3%) tumours were not measured. 193 (93%) tumours were unifocal while the remaining were multifocal (14, 7%). 91 (44%) cases demonstrated the presence of carcinoma in situ while the remaining 116 (56%) did not. 139 (67%) cases demonstrated the presence of lymphovascular invasion, 4 (2%) cases were indeterminate, and the remaining 64 (31%) did not demonstrate the presence of any lymphovascular invasion. 83 (40%) cases showed lymph node metastases while 115 (56%) did not; the remaining 9 (4%) cases lacked any lymph nodes. Soft tissue margins were positive in 52 (25%) cases. 7 (3%), 30 (15%), 111 (54%), and 59 (28%) cases were staged as pT1-pT4 respectively. 7 (3%), 22 (11%), 95 (46%), and 83 (40%) were staged as AJCC Stages 1–4, respectively.

### Immune microenvironment characterization

CD4, CD8, FOXP3, PD-1, and PD-L1 expression has been previously characterized in this cohort[1].

### CTA and MHC I characterization

**Fig 2:**
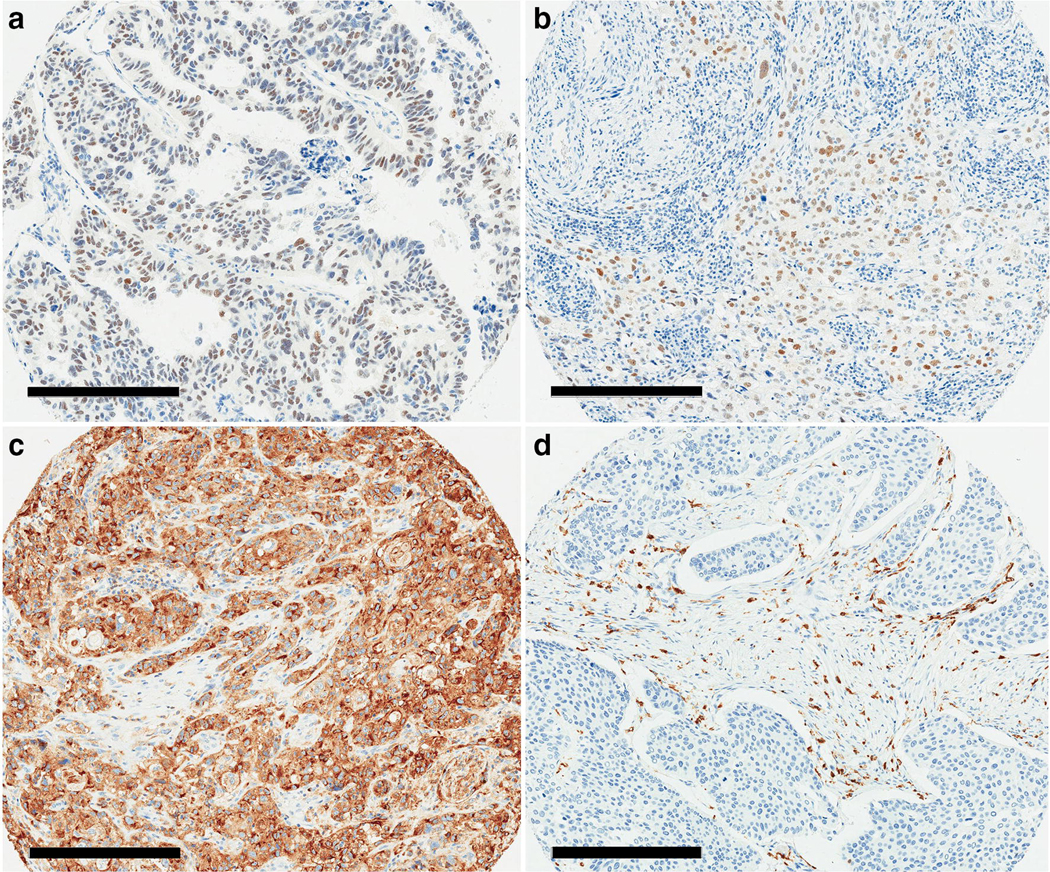
Cancer testis antigen and major histocompatibility complex I expression in high grade urothelial carcinoma. Expression of A. CT10 and B. PRAME in high grade urothelial carcinoma; note the nuclear expression in tumour cells and lack of expression in background stromal/inflammatory cells. C. Retained/normal MHC I expression, compared to D. complete lack of MHC I expression; note the retained expression in background stromal/inflammatory cells. Scale bar = 300 micrometers

CTA and MHC I expression shown in Table 1 and Figure 2. A total of 201 cases were evaluable for CT10 and PRAME expression: 42/201 (21%) and 31/201 (15%) of cases expression CT10 and PRAME, respectively. Both CT10 and PRAME expression was predominantly nuclear in location. There were no cases where the individual scores for triplicate cores were discordant (i.e. no cases where one core was scored as positive and the other ones as negative, or vice versa). Of note, CT10 and PRAME were co-expressed in 9/201 (5%) of cases.

A total of 202 cases were evaluable for MHC I expression: 25/202 (12%) cases demonstrated normal/retained expression, 18/202 (9%) cases demonstrated decreased/low expression, and the remaining 159/202 (79%) cases showed absent expression. There were no cases where the individual scores for triplicate cores were discordant.

### Correlation of immune microenvironment components with each other, neoadjuvant therapy status, and oncologic outcome

**Fig 3:**
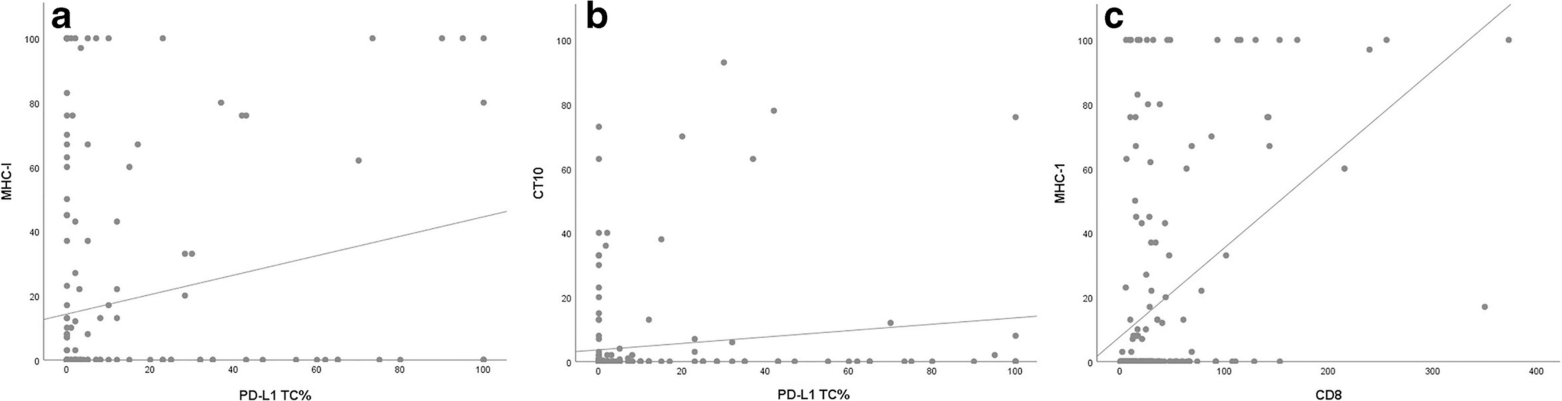
Correlation graphs depicting relationships between A. MHC I and PD-L1 in tumour cells, B. CT10 and PD-L1 in tumour cells, and C. MHC I and CD8

The Pearson correlation coefficients between the assessed immune parameters are shown in Table 2. Significant correlations ( p < 0.05) were noted for many immune parameter pairs including PRAME and FOXP3, PRAME and CT10, CT10 and PD-L1 in TCs and FOXP3, MHC I and PD-L1 in TCs, PD-L1 in ICs, PD-1, FOXP3, CD4, and CD8, PD-L1 in TCs and PD-L1 in ICs, PD-1, FOXP3, and CD8, PD-L1 in ICs and PD-1, FOXP3, CD4, and CD8, PD-1 and FOXP3, CD4, and CD8, FOXP3 and CD4 and CD8, and finally, CD4 and CD8. Figure 3 shows a graphical representation of some of the most significant correlations among the assessed immune parameters.

A total of 73 patients received some form of neoadjuvant therapy prior to cystectomy (31 with intravesical Bacillus Calmette-Guerin (BCG), 46 with chemotherapy/radiation; 4 patients received both BCG and chemotherapy/radiation). Of the 31 cases which were positive for PRAME, 15 (48%) were pretreated. Of the PRAME-negative cases, 58 (34%) were pretreated (p = 0.156, Fischer’s exact test). Of the 42 cases which were positive for CT10, 15 (36%) were pretreated. Of the CT10-negative cases, 58 (36%) were pretreated (p = 1.000, Fisher’s exact test). 3 of the pretreated cases (2 BCG, 1 chemotherapy/radiation) were positive for both PRAME and CT10. None of the cases which had been pretreated with both BCG and chemotherapy/radiation expressed were positive for either PRAME or CT10.

With regards to oncologic outcome, follow up data was available for 131 cases (median 4 months, range 1–206 months). Tumour recurrence was identified in 76/131 (58%) cases. 21/76 (28%) patients had metastatic disease. 24/131 (19%) patients were in palliative care at the time of last known follow-up. 4/24 patients in palliative care did not suffer from recurrence. 45/131 (34%) patients had died, 2 of which whose deaths were attributed to causes other than HGUC (myeloma/end stage renal disease and lymphoma, respectively).

**Fig 4:**
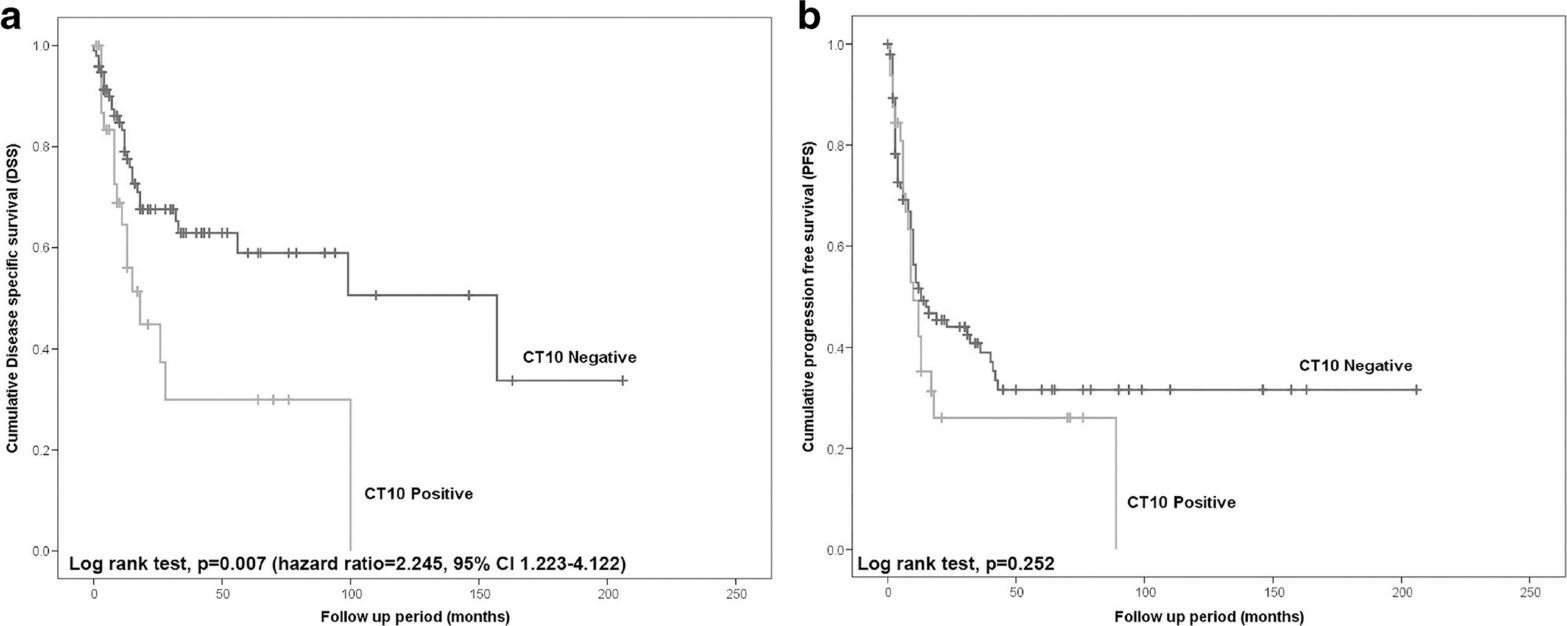
Kaplan-Meier survival curves comparing CT10 positive vs. negative tumours. Univariate analysis using log rank test showed that CT expression was significantly associated with worse A. disease specific survival, but was not associated with differences in B. progression free survival

Figure 4 shows the Kaplan-Meier survival curves (disease specific survival and progression free survival) comparing CT10 positive vs. CT10 negative tumours. With regards to DSS, CT10 positive tumours did significantly worse compared to CT10 negative tumours (log rank test p = 0.007, hazard ratio 2.245, 95% confidence interval 1.223–4.122). PFS did not differ significantly between CT10 positive and negative tumours (log rank test p = 0.252). PRAME and MHC I expression did not significantly affect DSS or PFS (log rank test p > 0.050).

## DISCUSSION

The tumour immune microenvironment is dynamic and complex and its evaluation has become increasingly important in a number of malignancies including HGUC of the bladder. Clinical trial data has shown checkpoint inhibition to be effective in the treatment of these variably aggressive malignancies[7–10] and because of this, there is increasing excitement regarding the possible use of other types of immunotherapies, including cancer vaccines, in the treatment of these and other tumours[11, 29–31]. CTAs are potential targets of cancer vaccines given that their expression is normally restricted to immune privileged sites with aberrant expression in cancer cells[17]. In this study, we identified CT10 and PRAME as cancer-associated antigens expressed in a subset of HGUCs. CT10 expression has been previously reported in bladder cancer[22] however, to the best of our knowledge, PRAME expression has not been previously studied in bladder cancer. Our assessment identified co-expression of CT10 and PRAME in a small proportion (5%) of our muscle-invasive HGUC cohort; the relevance and potential of this combinatory expression is as yet unclear. In a study which evaluated cases from a mixed stage cohort of HGUC, co-expression of CTAs was identified in up to 61% of cases[22]. The discrepancy in the number of co-expressed CTAs is likely explained by the fact that our cohort was evaluated with only two CTAs compared to Sharma *et al.*’s study which evaluated the expression of nine different CTAs. In addition, we utilised TMAs compared to whole tissue sections and our cohort is composed entirely of muscle invasive tumours whereas tumours ranging from pTa to pT4 were included in the aforementioned study.

Previous studies suggest that CTA expression by malignant cells is a poor prognostic factor, particularly in lung cancer[32–36] however, there are some discrepant reports in the literature[37–40]. As we have shown, CT10 expression was associated with significantly worse DSS in HGUC of the bladder. This is in contrast to the previously mentioned study by Sharma *et al.* (n= 94) which showed that patients with CT10 positive tumours had improved disease free and overall survival compared to patients with CT10 negative tumours[22]. As indicated above, the likely explanation for these differences is the difference in cohort composition, in addition to our cohort containing a much larger number of cases however, additional studies are needed to further evaluate this relationship

Importantly, a number of clinical trials are studying cancer vaccines in human malignancies and several have shown promising results in the treatment of bladder cancer[20, 41–43]. To the best of our knowledge, the relationship and correlation we have identified between CT10 and PD-L1 in TCs, although weak in this study, is novel. Of course, the clinical and therapeutic significance of this relationship will need to be further explored in future studies.

Of note, we did not identify any heterogeneity among CTA-expressing tumours in that all cases which were found to be positive for CT10 and/or PRAME expressed the antigen relatively equally throughout all evaluated TMA cores (both high and low expressing tumours). In addition, we did not observe any background expression in normal tissue elements (i.e. stromal cells, inflammatory cells). These findings are important, as they indicate that CTA assessment in small samples (i.e. biopsies) is reliable and a viable tool for evaluating CTA expression.

It is well known that antigen presentation by the MHC I-tumour antigen complex is a necessary step in the initiation of anti-tumour CD8+ T cell response[44]. Loss of MHC I expression has been recognized as a mechanism of tumour immune escape and there has been some discussion in the literature regarding possible MHC I recovery approaches, in order to improve tumour response to immunotherapeutic treatments[26, 45]. In our study, we show that MHC I expression correlates with many other cellular components of the immune microenvironment including CD8+ T cells. These relationships are intriguing and deserve additional study to determine their biological significance. Similar to PRAME, MHC I expression levels were not found to be significantly associated with worse DSS or PFS however, the differences may be significant in a cohort with more MHC-retained cases.

In conclusion, we have identified a number of interesting associations with regards to our characterization of the expanded tumour immune microenvironment in HGUC. Our understanding regarding the dynamics of the tumour immune microenvironment is evolving, and there is still much to be understood regarding the significance of correlations between different immune parameters. Further exploration of different components of the tumour immune microenvironment in a prospective multi-institutional cohort is warranted.

## Figures and Tables

**Table 1.** Cancer testis antigen (CT10, PRAME) and major histocompatibility class I (MHC I) expression in high grade urothelial carcinoma of the bladder.

	CT10 [n (%)]	PRAME [n (%)]
Positive	42 (21%)	31 (15%)
Negative	159 (79%)	170 (85%)
	A total of 201 cases assessed	
	MHC I [n (%)]	
Normal expression	25 (12%)	
Decreased/low expression	18 (9%)	
Absent Expression	159 (79%)	
	A total of 202 cases assessed	

**Table 2.** Pearson correlation coefficients for different immune parameters in high grade urothelial carcinoma.

	CT10	MHC I	PD-L1 TC	PD-L1 IC	PD-1	FOXP3	CD4	CD8
PRAME	**0.170**	−0.100	0.074	−0.024	0.050	**0.271**	0.024	−0.010
CT10		0.124	**0.153**	−0.040	−0.014	**0.284**	−0.064	0.080
MHC I			**0.214**	**0.235**	**0.162**	**0.171**	**0.299**	**0.449**
PD-L1 TC				**0.196**	**0.254**	**0.269**	0.099	**0.353**
PD-L1 IC					**0.604**	**0.379**	**0.593**	**0.631**
PD-1						**0.258**	**0.621**	**0.661**
FOXP3							**0.448**	**0.403**
CD4								**0.630**

MHC I: major histocompatibility complex I; programmed cell death ligand 1; program cell death 1; TC: tumour cells; IC: immune cells.

Bolded coefficients are significant (p < 0.050).
